# Kinins and Their Receptors as Potential Therapeutic Targets in Retinal Pathologies

**DOI:** 10.3390/cells10081913

**Published:** 2021-07-28

**Authors:** Rahmeh Othman, Gael Cagnone, Jean-Sébastien Joyal, Elvire Vaucher, Réjean Couture

**Affiliations:** 1 School of Optometry, Université de Montréal, Montreal, QC H3T 1P1, Canada; 2 Department of Pharmacology and Physiology, Faculty of Medicine, Université de Montréal, Montreal, QC H3T 1J4, Canada; 3 Department of Pediatry, Faculty of Medicine, CHU St Justine, Université de Montréal, Montreal, QC H3T 1J4, Canada; gael.cagnone@umontreal.ca (G.C.); js.joyal@umontreal.ca (J.-S.J.)

**Keywords:** kallikrein-kinin system, kinin receptors, diabetic retinopathy, age-related macular degeneration

## Abstract

The kallikrein-kinin system (KKS) contributes to retinal inflammation and neovascularization, notably in diabetic retinopathy (DR) and neovascular age-related macular degeneration (AMD). Bradykinin type 1 (B1R) and type 2 (B2R) receptors are G-protein-coupled receptors that sense and mediate the effects of kinins. While B2R is constitutively expressed and regulates a plethora of physiological processes, B1R is almost undetectable under physiological conditions and contributes to pathological inflammation. Several KKS components (kininogens, tissue and plasma kallikreins, and kinin receptors) are overexpressed in human and animal models of retinal diseases, and their inhibition, particularly B1R, reduces inflammation and pathological neovascularization. In this review, we provide an overview of the KKS with emphasis on kinin receptors in the healthy retina and their detrimental roles in DR and AMD. We highlight the crosstalk between the KKS and the renin–angiotensin system (RAS), which is known to be detrimental in ocular pathologies. Targeting the KKS, particularly the B1R, is a promising therapy in retinal diseases, and B1R may represent an effector of the detrimental effects of RAS (Ang II-AT1R).

## 1. Preface

Ocular pathologies involving chronic inflammation of the retina are particularly devastating in terms of visual acuity. Among these, age-related macular degeneration (AMD) and diabetic retinopathy (DR) are the leading cause of severe vision loss in the elderly and active population of industrialized countries, respectively. In addition to the chronic inflammation, vascular dysfunction and neovascularization, which correspond to the formation of new pathological branches from pre-existing retinal or choroidal vessels, occur. The inflammatory process includes a breakdown of the blood–retinal barrier, leukocyte adhesion on the blood vessel wall, macrophage and microglial activation, and cytokine and chemokine production. Current treatments of these diseases are only compensatory and consist commonly of invasive treatments such as quarterly intravitreal (ITV) injections of anti-angiogenesis agents (anti-VEGF antibodies) or laser coagulation to prevent loss of sight due to aberrant neovascularization. Moreover, a large population of patients does not respond to anti-VEGF therapy. To offer alternative and comfortable treatment to nonresponders, such as a topical approach, our team’s ongoing research effort has shown that the kallikrein-kinin system (KKS)—involved in inflammation—is overexpressed in the human AMD and DR retina and contributes to the development of pathological events in animal models of these diseases. Moreover, we were able to specifically target the KKS via topical ocular kinin B1 receptor (B1R) antagonist administration, which decreased neovascularization and retinal inflammatory responses. The purpose of this review is thus to better describe the possible involvement of the KKS in retinal diseases and therapeutical approaches that can prevent deleterious events that lead to blindness.

## 2. The kallikrein-kinin system

**Figure 1:**
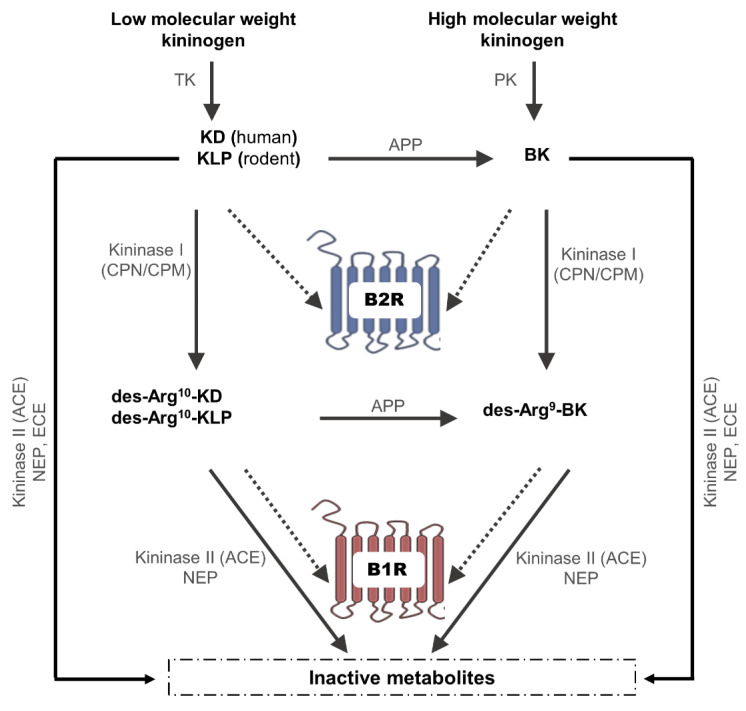
Biosynthesis and metabolism of kinins. Low- and high-molecular-weight kininogens are cleaved by tissue kallikrein and plasma kallikrein, respectively, into kallidin (in humans) and kallidin-like peptide (in rodents) [5,6], and bradykinin. Bradykinin, kallidin, and kallidin-like peptide are then either converted by the action of kininase I to des-Arg^9^-bradykinin, des-Arg^10^-kallidin, and des-Arg^10^-kallidin-like peptide, respectively, or inactivated by kininase II, neutral endopeptidase 24.11 (neprilysin, NEP), and the endothelin-converting enzyme [4,7,8,9,10,11]. ACE, angiotensin-1- converting enzyme (also known as kininase II); APP, aminopeptidase P; B1R, bradykinin type 1 receptor; B2R, bradykinin type 2 receptor; BK, bradykinin; ECE, endothelin-converting enzyme; KD, kallidin; KLP, kallidin-like peptide, which is Arg(1)-kallidin (Arg(0)-bradykinin); NEP, neutral endopeptidase; PK, plasma kallikrein; TK, tissue kallikrein.

The kallikrein-kinin system (KKS) is a complex multi-enzymatic and peptidergic system known to play a critical role in human physiology, but also in pain and inflammation [1,2,3]. Its physiological functions encompass nociception, cardiovascular and renal functions, vasomotricity, and host defense to infectious diseases [2,4]. The KKS is constituted by a panel of vasoactive peptides (kinins), synthesized and metabolized by different enzymes (kallikreins and kininases), and two G-protein-coupled receptors (GPCR) (Figure 1).

### 2.1. Kinins Generation

Kinins are small peptides of 9–11 amino acids, including bradykinin (BK), kallidin (KD or Lys-BK), kallidin-like peptide (Arg(1)-kallidin (Arg(0)-bradykinin)), and T-kinin (Ile-Ser-BK; expressed exclusively in rats), which are generated from high-molecular-weight kininogen (88 to 120 kDa) (HK) and low-molecular-weight kininogen (50 to 68 kDa) (LK) under the action of plasma kallikrein (PK) and tissue kallikrein (TK) [4,5,6]. BK generation in plasma takes part in the intrinsic coagulation pathway activation, involving the interaction of Factor XII (Hageman factor), prekallikrein (PPK), and Factor XI with HK on negatively charged surfaces, such as components of the extracellular matrix or other negatively charged particles (cholesterol sulfate, urate, or phospholipid acid), leading to prothrombotic and inflammatory effects [2,12]. Aminopeptidase P transforms KD and KLP into BK, while kininase I that includes carboxypeptidases N (CPN) and M (CPM) transforms BK, KD, and KLP into des-Arg^9^-BK, lys-des-Arg^9^-BK (or des-Arg^10^-KD), and des-Arg^10^-KLP, respectively. Alternatively, kininase II (also called angiotensin-1-converting enzyme (ACE)), neutral endopeptidase 24.11 (neprilysin, NEP), and the endothelin-converting enzyme (ECE) degrade BK, KD, and KLP into inactive fragments on the canonical B1 and B2 receptors [4,7,8,9,10,11]. Moreover, ACE and NEP can metabolize des-Arg^9^-BK, des-Arg^10^-KD, and des-Arg^10^-KLP into inactive metabolites. It is worth noting that the enzymes involved in the catabolism of kinins are also involved in the metabolism of other peptides belonging to other systems such as angiotensin, endothelin, anaphylatoxins C3a and C5a, substance P, neurotensin, enkephalins, atrial natriuretic peptides, and chemotactic peptide [5,8,13,14,15,16,17,18,19,20].

### 2.2. Kinin Receptors

The KKS operates through the activation of two GPCR, bradykinin type 1 (B1R), and type 2 (B2R) receptors. While BK, KD, and KLP are the endogenous agonists of B2R, their kininase I metabolites (deprived of C-terminal Arginine) are the preferred agonists of B1R [3,21]. The agonist selectivity of mouse B1R differs from human and rabbit B1R; des-Arg^9^-BK is the preferred B1R agonist in mice, while des-Arg^10^-KD displays much higher selectivity for human and rabbit B1R [22]. B2R can activate a plethora of signaling pathways either indirectly by interacting with guanine nucleotide-binding proteins, mainly with Gαq and less commonly with Gαs, Gαi, and Gα_12/13_, as reviewed in [23]; or directly by interacting with endothelial nitric oxide synthase (eNOS), phospholipase A 2 (PLA2), and tyrosine phosphatase (SHP2) [4]. B1R interacts with the same guanine nucleotide-binding proteins as those of B2R, but preferentially with Gαq to activate the phosphatidyl inositol-mitogen-activated protein kinase (MAPK) pathway, and with Gαi to activate the extracellular signal-regulated kinase (ERK)-inducible nitric oxide synthase (iNOS) pathway [4,23,24].

Most physiological effects of kinins are mediated by the constitutive B2R, since B1R is virtually absent in healthy tissues. BK is a potent endothelium-dependent vasodilator that has important cardiovascular and renal functions via the B2R [25]. Moreover, B2R contributes to the therapeutic effects of angiotensin-1-converting enzyme inhibitors (ACEI) and angiotensin AT1 receptor blockers [26]. These benefits derive primarily from its vasodilatory, antiproliferative, antihypertrophic, antifibrotic, antithrombic, and antioxidant properties [4,26,27,28,29,30,31,32,33,34,35,36]. However, it is worth noting that B2R can also contribute to inflammation. Indeed, uncontrolled production of kinins and excessive activation of B2R may lead to unwanted pro-inflammatory side effects as observed in angioedema, septic shock, stroke, hypertension, and Chagas vasculopathy, in which B2R antagonism is salutary [4,26,37,38,39,40,41].

B1R, however, is induced and upregulated during tissue injury involving the cytokine pathway, oxidative stress, and the transcriptional nuclear factor NF-κB [2,38,39,42,43,44]. The highly inducible character of B1R is often symptomatic of the occurrence of autoimmune, infectious, cardiac, kidney, and bowel inflammatory diseases [2,45,46,47,48,49]. However, B1R may play a compensatory role for the lack of B2R, and its upregulation during tissue damage may be a useful mechanism of host defense [25,50,51,52].

### 2.3. Kinin Receptors in Inflammation and Neovascularization

B1R antagonism or deletion plays a protective role in inflammation, organ damage, and lethal thrombosis in septic shock in diabetes [53]; lipopolysaccharide (LPS) mediated acute renal inflammation [54]; renal ischemia-reperfusion injury [55]; and in cardiovascular [56] and retinal [57,58,59,60] inflammatory diseases. B1R inhibition reversed vascular [61] and retinal [58,60] inflammation induced by diabetes mellitus. Moreover, genetic deletion of B1R or administration of B1R antagonist in mice reduced pro-inflammatory mediators’ expression and increased anti-inflammatory mediators [55]. Besides the well-described pro-inflammatory roles of both kinin receptors, an anti-inflammatory effect has been attributed to B2R. For instance, intramyocardial injections of tissue kallikrein reduced the expression of many inflammatory mediators through B2R activation [62]. Moreover, BK can counteract the inflammation in the brain [63]. Indeed, BK reduced LPS-induced TNF-α release from microglia activated by B1R [63]. Recently, a neuroprotective role for B2R was highlighted, and the use of B2R agonists was proposed as a possible therapeutic option for patients diagnosed with Alzheimer’s disease [64]. Altogether, these findings support a dual role of B2R in inflammation, whereas B1R is mainly involved in the inflammatory responses, especially those triggered by cytokines or pathogens [65,66,67]. Because B1R is a potent activator of iNOS and NADPH oxidase, it is associated with vascular inflammation, increased vascular permeability, insulin resistance, endothelial dysfunction, and diabetic complications [24,43,44,68,69,70].

The contribution of kinin receptors to neovascularization has been widely studied in various models and diseases. In some vascular diseases, drugs are used to inhibit neovascularization (i.e., cancer, neovascular retinal pathologies, etc.), while in others such as ischemia, treatments aim to stimulate neovascularization. Therefore, both activation and inhibition of kinin receptors are important drug targets of vascular diseases. For instance, the activation of B1 and/or B2 receptors may be beneficial, notably in neovascularization and angiogenesis in diabetic mice, renal ischemia/reperfusion injury, diabetic nephropathy, and cerebral and heart ischemia [38,71,72,73,74,75,76,77,78]. B1R deletion or antagonism was shown to impair neovascularization, while B1R agonist had a positive outcome in a model of hindlimb ischemia in diabetic mice [77]. In the same model, B1R or B2R agonists administration induced revascularization by stimulating the mobilization of monocytes and proangiogenic CD34/VEGFR-2 mononuclear cells, and the infiltration of macrophages [76]. Moreover, B1R inhibition prevented the revascularization, as well as VEGF, eNOS, and basic fibroblast growth factor (FGF2) upregulation, induced by ACE inhibitor [79]. While the proangiogenic effect of ACE inhibitor was attributed to an increase in BK generation (Figure 1) and the activation of B2R in diabetic ischemia [80], B1R was more implicated than the B2R in ACE inhibitor mediated angiogenesis in Ang II type 1a receptor knockout (AT1aKO) mice after hindlimb ischemia [81]. Indeed, the B1R antagonist reversed the neovascularization and reduced VEGF-A and VEGFR-2 expression, while the B2R antagonist had less impact [81].

Cancer is among the diseases for which inhibiting kinin receptors would be beneficial. Indeed, the role of kinin receptors in promoting angiogenesis was supported by many experimental studies using cancer cells/tissues. For instance, B1R activation was shown to increase IL-4 and VEGF generation from human keratinocytes and to stimulate endothelial cell migration, thus promoting neovascularization [82]. Furthermore, when human endothelial cells were co-cultured with neuroblastoma cells, B1R and B2R expression was observed at the sites of interaction between these two cell types, regulating angiogenesis and tumorigenesis [83]. Interestingly, blockade of either B1R or B2R reduced tumor vascularization in vivo and significantly inhibited proliferation and migration of colorectal cancer cells in vitro [84]. In studies of mice bearing sarcoma 180 cells, it was suggested that BK promotes angiogenesis in the early phase of tumor development by increasing vascular permeability via B2R, expressed in the endothelial cells and not via B1R, and in the late phase by stimulating the upregulation of VEGF via B2R in the stromal fibroblasts [85,86,87]. BK was also found to increase VEGF expression in human prostate cancer cells and further promote tumor angiogenesis. Interestingly, B2R blockade using antagonists or genetic deletion reduced VEGF expression and abolished prostate cancer cell conditional medium-mediated angiogenesis [88]. Altogether, these studies suggest that kinins play a pivotal role in angiogenesis through B1R, B2R, or both.

The dual beneficial and deleterious effects of kinin receptors raise questions about the therapeutic value of B1R/B2R agonists or antagonists in various diseases. Hence, the Janus face of kinin receptors needs to be seriously addressed in each pathological setting. The discovery of the expression of kinin receptors and other KKS components in the eye led many investigators to address their physiological and pathological roles, particularly in the retina.

## 3. kallikrein-kinin system in the Eye

Similarly to other organs, the KKS in the eye is a double-edged sword, as it contributes to many physiological processes including blood-flow regulation and vascular tone control, but also partakes in the complex processes of inflammation [4,57,89]. It was reported that the KKS underlies a number of ocular pathologies (DR, AMD, choroidal neovascularization, macular edema) associated with inflammation and pathological neovascularization, particularly in the human and rat retina [57,58,59,60,69,90,91,92,93]. For instance, PK and HK, by binding to the vascular endothelium, release BK and subsequently activate B2R, which plays a key role in the control of vascular tone [4]. However, in diabetic rats, an increase in PK mediates retinal vascular dysfunction and induces retinal thickening [91]. Moreover, tissue kallikrein (TK) was expressed in the human retina, cornea, and ciliary body [94]. TK does not seem to be implicated in retinal pathologies, particularly in diabetic retinopathy, as it was slightly detectable in vitreous fluids of patients with severe proliferative DR [95]. An expression of TK, B1R, and B2R was also reported at multiple tissue sites in the anterior portion of the human eye [96]. Nevertheless, B2R but not B1R was expressed in the control human retinae [93]. BK produces B2R-mediated vasodilatation of retinal vessels in control rats [97]. This response involves the COX-2 pathway, including prostacyclin [97]. Hence, B2R contributes to retinal blood flow control. On the other hand, the vasodilatation mediated by kinins is associated with B2R and B1R in streptozotocin (STZ)-diabetic rats and involves both NO and prostacyclin [97]. A protective compensatory role on retinal microcirculation was attributed to B1R at day 4 but not at 6 weeks following diabetes induction [98]. Likewise, both B1R and B2R contribute to the increased retinal vascular permeability in STZ-diabetic rats [58,60,99]. Collectively, these studies support the presence of the KKS throughout the eye and its ability to influence ocular function in health and disease.

It is still unclear whether the KKS expression is generated locally in the eye, or if it is a result of a systemic infiltration of KKS components. While the observation of some KKS components in the healthy eye [94] suggests a local production of these components, Phipps and Feener have suggested an infiltration of these components from the systemic circulation that could happen in DR [100]. This was explained by the increase of KKS components expression in the plasma of diabetic patients, and their infiltration in the retinal interstitium and vitreous that may occur following the increase in vascular permeability and hemorrhages in the retinal vessels [100]. Nonetheless, whether the origin of the KKS expression is local or a result of its infiltration from the systemic circulation in the eye, all these studies support an implication of the KKS in the pathogenesis and development of retinal diseases, such as DR and age-related macular degeneration (AMD).

## 4. kallikrein-kinin system in Diabetic Retinopathy

DR is one of the most common microvascular complications of diabetes, observed in up to 90% of patients with type 1 diabetes and 50 to 60% of patients with type 2 diabetes, despite a tight glycemic control [101,102,103]. If left untreated, DR can cause severe vision loss. Current therapeutic strategies target the advanced stages of the disease and aim to slow its progression without really reversing its outcome [104]. Among the current treatments for proliferative DR and macular edema are laser photocoagulation, vitrectomy, and intravitreal injections of corticosteroids or anti-VEGF that could prevent further vision loss [105]. However, the curative activity of these treatments is limited by side effects. For instance, pan-retinal photocoagulation can cause a loss of peripheral vision, color vision, and night vision [106]; intravitreal injection of anti-VEGF has a short effect duration and can cause a tractional retinal detachment and endophthalmitis [107], and many patients are refractory to it [108,109,110]. Importantly, there is no effective treatment for the highly widespread early stages of the disease [111]. Thus, there is an urgent need for less-invasive and more-effective therapeutic strategies.

### Kallikreins and Kinin Receptors in Diabetic Retinopathy

A decrease in the concentration of kallikrein-binding protein (KBP), a serine protease that binds to tissue kallikrein and inhibits kallikrein activity, was reported in the vitreous humor of patients with proliferative DR [112]. Parallel to this study, the levels of KBP were reported to be decreased by 60% for at least 4 months in the retina of STZ-diabetic rats [113]. Moreover, tissue kallikrein was significantly elevated in vitreous fluid in proliferative DR patients when compared with control patients [114]. Interestingly, intravitreal injection of kallistatin, a tissue kallikrein inhibitor, in STZ-diabetic rats reduced retinal neovascularization; however, these effects have been attributed to the tissue kallikrein effects on the VEGF system [115]. Other components of the plasma KKS, including PK, FXII, and HK were also found in the vitreous fluid of patients with advanced DR [116,117]. Increased levels of PK and PK activity were observed in the retina of diabetic rats compared with nondiabetic controls [90,91]. PK injection increased vascular permeability in the healthy retina, and further in the diabetic retina, yet these effects were reversed by the inhibition of PK [91]. Furthermore, the retinal thickening, as well as the increase in vascular permeability caused by intravitreal injections of VEGF, were reduced (by 47% and 68%, respectively) in plasma prekallikrein knockout mice [118]. In phase I.B of a recent clinical trial, PK inhibition by one-time intravitreal injection of KVD001 was shown to be effective in treating macular edema without creating a safety concern. The injection improved visual acuity and central retinal thickness, and no exacerbation of the severity of DR was observed [119]. PK contribution to DR pathogenesis was, however, attributed to B2R activation. Indeed, C1 inhibitor-deficient mice caused vasogenic edema due to increases in PK expression, BK synthesis, and activation of B2R [120]. Given the fact that PK is a constitutive enzyme involved in other systems, including thrombosis and blood hemostasis, its inhibition may risk interfering with its physiological role [57].

Alternatively, B1R expression was shown to be significantly increased in retinae of rats and humans affected by type 1 and type 2 diabetes [57,58,60,69,92]. B1R expression was enhanced on the 4th day of STZ-diabetic retina [97], and it remained upregulated even 6 months after the induction of type 1 diabetes [58]. B1R upregulation in STZ-diabetic rats leads to retinal microvessel vasodilation [97], vascular hyperpermeability, and inflammation [60]. Importantly, these responses were reversed by eye-drop application of B1R antagonists (LF22-0542 and R-954) [58,60]. B1R was strongly expressed in vascular endothelial cells and in the retinal pigment epithelium of human and rats’ retinae, suggesting its implication in altering the integrity of the internal and external blood–retinal barrier (BRB) in DR and AMD [58,59,69,92,93]. B1R might disrupt the BRB [59,60,69] either by the suppression of tight junction components (occludin, claudin, and zonula occludens-1), or by a rearrangement of the cytoskeleton filamentous actin [121]. In human cerebral microvascular endothelial cells, B1R agonist (des-Arg^9^-BK) was shown to decrease the expression of zonula occludens-1 and occludin in vitro [122]. Altogether, these data support a strong implication of B1R in DR.

One additional mechanism by which B1R contributes to the pathogenesis of DR has been recently suggested that involves the activation of the iNOS pathway [69]. In HEK293 cells, it was shown that B1R associated with Gαi can activate iNOS through ERK [24,123], thereby producing sustained amounts of NO. Interestingly, iNOS inhibition in the retina of diabetic mice caused a decrease in occludin and zonula occludens-1 expression, thus protecting the dissociation of BRB [124]. The elevated concentrations of NO, nitration of proteins, prostaglandin E2, superoxide, leukostasis, and retinal thickness induced by diabetes were significantly inhibited in diabetic iNOS (−/−) mice [125]. In addition, diabetes-induced acellular capillaries and pericyte ghosts were significantly inhibited in diabetic iNos (−/−) mice [125]. Given that B1R can also enhance the production of superoxide anion through PLC and the activation of NADPH oxidase [44], NO produced by iNOS upon B1R activation can react with superoxide anion to yield peroxynitrite, a highly toxic molecule [126,127,128], which causes endothelial and neuronal cell apoptosis, neuronal degeneration, and BRB breakdown in DR [125,126,129,130,131,132,133]. Peroxynitrite can also activate NF-κB, and thereby can increase the expression of several pro-inflammatory mediators, including B1R [4,43]. Hence, B1R activation can further amplify and perpetuate the inflammatory response, as well as the oxidative stress, through a positive feedback loop [43,69] In resonance with this hypothesis, pharmacological iNOS inhibition in the retina of STZ-diabetic rats reversed peroxynitrite formation, the upregulation of inflammatory mediators (notably B1R), and the enhanced vascular hyperpermeability induced by B1R agonist [69]. Collectively, these data support a robust implication of the B1R in DR, mainly by increasing and perpetuating inflammation and oxidative stress. Hence, targeting B1R represents a promising therapeutic approach in DR, and deserves further investigation.

**Figure 2:**
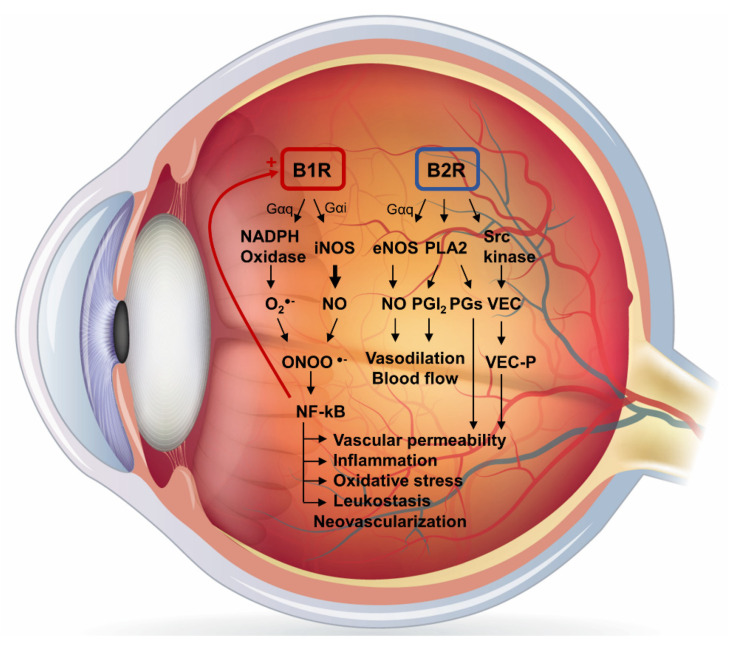
kallikrein-kinin system in diabetic retinopathy. Schematic proposal of the signaling pathways activated by B1R and B2R in diabetic retinopathy. PGs, prostaglandins; PGI2, prostacyclin; PLA2, phospholipase A2; Src, kinase proto-oncogene tyrosine-protein kinase; VEC, vascular endothelial cadherin; VEC-P, phosphorylated vascular endothelial cadherin. The human eye anatomy diagram was acquired from Shutterstock (http://www.shutterstock.com, accessed on 16 July 2021).

In the retina of 2-week STZ-diabetic rats, B2R mRNA and protein expression did not change when compared to the control retina [69], yet a significant increase in B2R mRNA was observed at 24 weeks in the retina of diabetic rats [58]. B2R contributes to the increased retinal vascular permeability in STZ-diabetic rats [99]. Indeed, BK induces vascular endothelial cadherin phosphorylation and a subsequent rapid internalization and ubiquitination, leading to an opening of endothelial cell junctions and plasma leakage [134] (Figure 2). However, more studies are needed in DR using recently developed selective and stable B2R antagonists or biostable kinin analogs [41,46,135]. 

## 5. kallikrein-kinin system in Age-Related Macular Degeneration

AMD is a multifactorial disorder, highly heritable, and caused by an interplay of many factors including age, genetic, and environmental risk factors. The prevalence of AMD is rising worldwide, and it is expected to increase from 196 million in 2020 to 288 million by 2040 [136,137]. In its early stages, AMD is characterized by pigmentary abnormalities and deposits of lipoproteinaceous debris (soft drusen) between the basal lamina of the retinal pigment epithelium (RPE) and the inner collagenous layer of Bruch’s membrane (BM) of the central retina [138]. Early and late forms of AMD include wet (exudative) AMD and dry (nonexudative) AMD. The late form of dry AMD is also called geographic atrophy AMD. Exudative/wet AMD is mainly characterized by neovascularization that arises from the choroid, but in about 10–15% of the cases, originates from the retinal vasculature in the subretinal space [139,140]. Dry AMD is more prevalent, affecting 85–90% of patients suffering from AMD [136,137], and is characterized by an extending lesion of the RPE and photoreceptors [141]. Current treatments target only the neovascular AMD mainly by anti-angiogenic therapy (anti-VEGF), which aims to decrease the vascular permeability and to inhibit the formation of new vessels without treating the degenerative processes and the vision loss in 30% of patients that occur in the long term [142]. On the other hand, no effective treatment options are available for dry AMD, besides lifestyle modification and nutrient supplementation [143].

Similarly to DR, the pathogenesis of AMD is driven by both inflammation and microvascular alterations leading to BRB dysfunction and pathological neovascularization. Indeed, an increase of diverse transcriptional factors (NF-kB, HIF-1α) and pro-inflammatory mediators (cyclooxygenase-2 (COX-2) products, IL-1β, TNF-α, iNOS, NO) has been reported in different models of DR and AMD [58,59,60,69,144]. Consistently with the roles of kinin receptors in both inflammation and neovascularization, we showed that most upregulated inflammatory mediators were blocked by B1R inhibition in DR and AMD [58,59,60]. B1R was shown to be expressed on Müller cells and astrocytes in these retinal pathologies in rat and post-mortem human retina [58,59,93], and on microglia in post-mortem human wet AMD retina [93]. Macroglia play a primary role in vascular function and neuronal integrity of the retina [145]. These results deserve closer scrutiny and encourage further investigations to assess the impact of an ocular treatment with a B1R antagonist on macro- and microglial reactivity in DR and AMD.

B1R expression was upregulated in a rat model of choroidal neovascularization (CNV), and B1R blockade reduced the size of the neovascularization [59]. B1R contribution to retinal neovascularization in humans was also suggested in a recent study in post-mortem human wet AMD retinae. In these retinae, B1R was strongly expressed in endothelial/vascular smooth muscle cells, and co-localized with iNOS and fibrosis markers. Its presence on vascular smooth muscle cells can induce prolonged vessel constriction and consequently contribute to retinal ischemia, a main trigger of neovascularization, mainly by activating the VEGF-A pathway [146]. Altogether, these data highlight a contribution of B1R to retinal pathologies associated with neovascularization. By analogy with another ocular pathology, B1R agonist administration in the rabbit eye induced corneal neovascularization, an effect that was reversed by B1R inhibition with the same efficacy as VEGF-A inhibition [147]. The implication of B2R in ocular neovascular pathologies has also been suggested. For instance, in an ischemic retinopathy model, B2R antagonist (Fasitibant) significantly decreased the expression of VEGF and FGF2, as well as pathological retinal neovascularization [148]. In a mice model of CNV, B2R blockade with Icatibant had a limited effect, yet concomitant inhibition of B2R and kininase II had additive suppression of the CNV size [149]. We reported no significant modification of B2R mRNA and protein expression in human neovascular AMD retinae [93].

**Figure 3:**
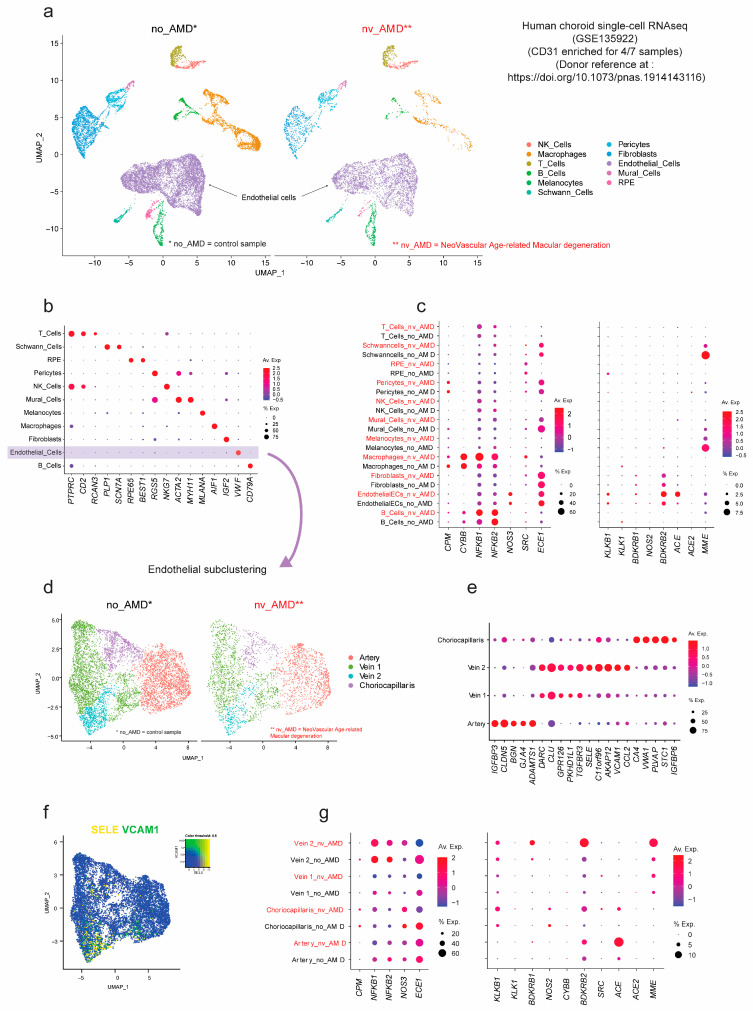
Transcriptomic impact of age-related macular degeneration on the kallikrein-kinin system in human choroid tissues by single-cell RNA seq. (**a**) Dimensionality reduction and cluster visualization with UMAP plot. Color-coded clusters represent the different choroid cell types (see legend in bottom right corner) identified by single-cell RNAseq analysis of post-mortem control (left panel) and neovascular AMD (right panel) choroids (public data deposited on GEO with reference number GSE135922). (**b**) Dotplot of the expression of the gene markers used to identify choroid cell types. (**c**) Dotplot of the expression of the genes involved in the KSS pathway across choroidal cell types from control (black legend) and neovascular (nv)-AMD (red legend) samples. As shown in (**a**–**c**), KLKB1, BDKRB1, and BDKRB2 are mainly expressed in choroidal endothelial cells, albeit at low expression levels; some expression was also detected in fibroblasts. (**d**) Dimensionality reduction and cluster visualization with UMAP plot of the subpopulations of choroid endothelial cells of post-mortem control (left panel) and neovascular AMD (right panel) choroids. Choroid cells clustered into four distinct endothelial cell subtypes: two venous subtypes, one choriocapillaris subtype, and one arterial subtype (see legend on right side). (**e**) Dotplot of the expression of the specific gene markers in these endothelial subcluster, as annotated by Voigt et al., [150]. (**f**) Visualization with UMAP plot of E SELECTIN (SELE) and VCAM1 expression co-localizing to vein 2 subcluster, a signature reminiscent of post-capillary venous identity. (**g**) Dotplot of the expression of genes involved in the KSS pathway across choroidal endothelial cell subtypes from control (black legend) and neovascular (nv)-AMD (red legend) samples. Vein cluster 2 showed greater expression of KKS genes, notably BDKRB1, BDKRB2, and MME, across all choroidal endothelial cells of control and nv-AMD choroid samples. In all the dotplots, the size of the dots encodes the percentage of cells within a class, and the color scale encodes the average expression level across all cells within a class (red being the strongest value). Av. Exp., average gene expression across all cells within each cluster; % Exp., percentage of cells with detectable gene expression within each cluster.

In addition to KKS gene expression in the ocular pathologies reviewed above, we also mined a recent public single-cell transcriptomics database of post-mortem choroid tissues from neovascular AMD human patients [150], using previously described analyses [151,152]. KKS genes were detected in fibroblasts and immune, RPE, and endothelial cells (Figure 3, unpublished original results). Choroidal endothelial cell specifically expressed *KLKB1*, *BDKRB1,* and *BDKRB2* (genes for prekallikrein, B1R, and B2R, respectively), albeit at low expression levels. Subclustering of the heterogenous choroidal endothelial cell population identified four subtypes (see legend of Figure 3), including vein clusters 1 and 2, discriminated by the higher expression of *SELECTIN E* and *VCAM1* (Figure 3f), a pattern reminiscent of post-capillary venous identity [153]. Interestingly, vein cluster 2 showed greater expression of KKS genes, notably *BDKRB1*, *BDKRB2,* and *MME* (genes for B1R, B2R, and neprilysin, respectively) in choroid endothelial cells from AMD patients (Figure 3g). Although the relatively low detection levels for these three genes (less than 10%) requires cautious interpretation, their specific expression in post-capillary venous endothelial cells of neovascular AMD patients is intriguing and warrants further investigation of kinin receptors in AMD.

Although recent studies support the implication of the KKS in wet neovascular AMD, it is still not clear if the KKS is implicated in the dry form. In the retina of aged rats, an increase of KKS components was demonstrated, where 4-month-old rats showed a significant decrease in KBP, and consequently an increase in tissue kallikrein compared to 2-week-old rats [113]. Recent data using post-mortem human retinal sections showed only a weak expression of B1R and no changes of B2R in dry AMD [93].

## 6. kallikrein-kinin system in Other Retinal Damage

This review highlights the implication of the KKS in retinal pathologies associated with inflammation and neovascularization. However, KKS can also be implicated in ocular pathologies such as glaucoma and ocular ischemia. For instance, BK alters the shape of cells in both bovine and human trabecular meshwork [154,155,156]. Moreover, FR-190997, a B2R agonist, was shown to lower the intraocular pressure by promoting uveoslceral outflow in monkeys [157]. Taken together, these results suggest that the KKS can also be implicated in ocular diseases with elevated intraocular pressure. Intravenous administration of TK protected against retinal ischemic damage in a retinal ischemia/reperfusion model in mice [158]. In this model, TK administration inhibited retinal ganglion cell death, counteracted the retinal permeability induced by ischemia, and improved the visual function [158]. However, these protective effects seem to be independent of blood flow and might be mediated by eNOS activation and subsequent NF-κB silencing.

## 7. Crosstalk between the kallikrein-kinin system and the Renin–Angiotensin System (RAS) in Ocular Pathologies

**Figure 4:**
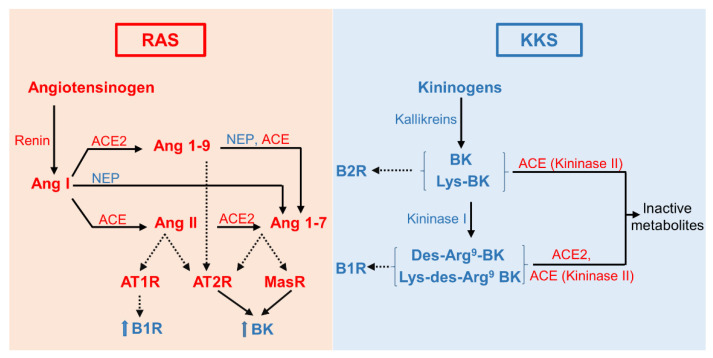
Crosstalk between the kallikrein-kinin system (KKS) and the renin–angiotensin system (RAS). ACE, angiotensin -1 -converting enzyme (kininase II); ACE2, angiotensin-converting enzyme 2; Ang I, angiotensin I; Ang II, angiotensin II; AT1R, angiotensin type 1 receptor; AT2R, angiotensin type 2 receptor; BK, bradykinin; MasR, Mas receptor; NEP, neutral endopeptidase 24.11 (neprilysin, NEP).

There is compelling evidence for a local renin–angiotensin system (RAS) within the human eye that is activated in ocular disorders and DR [159,160,161,162,163]. Multiple interactions (crosstalk) exist between the RAS and the KKS [4,25,164,165,166] (Figure 4). In addition to the implication of ACE (kininase II) in the degradation of kinins (acting on B1R and B2R) and the formation of angiotensin II (Ang II) from angiotensin I (Ang I) [165], the activation of the angiotensin II type 2 receptor (AT2R) leads to BK generation, which promotes vasodilation through the NO/cGMP system [167]. Under the action of angiotensin-converting enzyme 2 (ACE 2), Ang I is cleaved into angiotensin-(1-9) (Ang-(1-9)), a peptide that elicits vasodilation and anti-inflammatory effects through activation of AT2R [168,169]. ACE 2 can also cleave Ang II to Ang-(1-7), an agonist of AT2R and Mas-receptor (MasR) that elicits the release of BK, vasodilatory, antiproliferative, anticoagulation, anti-inflammatory, and antifibrotic activity, thus counterbalancing the adverse effects of Ang II mediated by AT1R [170,171,172]. Importantly, ACE 2 hydrolyses B1R agonists (des-Arg^9^-BK and Lys-des-Arg^9^-BK) into inactive metabolites and therefore impairment of ACE 2 (as under COVID-19 infection) is expected to enhance the pro-inflammatory effects of the des-Arg^9^-BK/B1R axis [164,173] (Figure 4). Moreover, the pro-inflammatory effects of Ang II was attributed to AT1R and B1R activation [174]. Following AT1R activation, Ang II enhances B1R expression in vitro [175,176] and in vivo [174,177] by activating NADPH oxidase, IL-1β, IL-6, TNFα, and NF-κB [174,176]. Besides ACE2, neutral endopeptidase 24.11 (NEP) was described to be biochemically capable of producing Ang-(1-7) from Ang I and Ang-(1-9) [178]. NEP can also hydrolyze Ang-(1-7) to form angiotensin-(1-4) (Ang-(1-4)), an inactive metabolite [179]. Hence the reciprocal interaction between the RAS and the KKS must be considered in the development of novel therapeutic approaches in the treatment of retinal diseases.

### 7.1. Renin–Angiotensin System in Diabetic Retinopathy

The RAS is implicated in inflammation, vascular alterations, neovascularization, and edema in retinal pathologies, notably in DR and retinopathy of prematurity [163]. An increase in prorenin level was reported in the vitreous fluid of patients with proliferative DR [161]. Ang II induces pericyte apoptosis in the retina in vivo and in vitro in hypertensive rats by increasing the expression of RAGE receptor for advanced glycation end products (AGEs); these effects were reversed by an Ang II-AT1R blocker [180]. An AngII-AT1R blocker (Candesartan) inhibits the development of DR by reducing the accumulation of AGEs and the expression of VEGF in the retina in a rat model of type 2 diabetes [181]. This AT1R blocker reduces retinal vascular permeability induced by diabetes and Ang II in rats [182]. Importantly, the DIRECT study based on more than 1400 patients found that Candesartan reduces the progression of microaneurysms in both type 1 and type 2 diabetic patients, yet no effects were observed on the DR regression and progression, or on the prevention of diabetic macular edema risk [183]. Another multicenter study of 285 patients with type 1 diabetes reported that Losartan, another AT1R blocker, slows the progression of DR [184]. Together, these studies suggest that angiotensin AT1R blockers may be effective against DR independently of their anti-hypertensive action.

ACE inhibition was also shown to lower the risk and prevent the development and the evolution of DR in humans [185,186]. ACE inhibition reduces retinal VEGF overexpression and hyperpermeability in experimental diabetes [187] and vitreous VEGF concentrations in patients with proliferative DR [188]. Interestingly, changes in circulating VEGF do not account for the beneficial effect of ACE inhibition on retinopathy in patients with type 1 diabetes [189]. Previous clinical trials have associated the decrease in DR progression in type 1 or type 2 diabetic patients with a reduction of hypertension [190,191]. The United Kingdom Prospective Diabetes Study (UKPDS) with more than 1000 patients reported a reduction in the progression of DR with ACE inhibitor and β1-adrenergic receptor blocker, suggesting that the beneficial effect may be related to the anti-hypertensive and not to the ACE-inhibition-specific effect [190]. Nevertheless, other studies have reported a slowdown in DR progression in normotensive diabetic patients taking an ACE inhibitor, suggesting a possible therapeutic effect of ACE inhibitors not related to the anti-hypertensive effect [184,191]. In resonance with this, a meta-analysis of 21 clinical trials with more than 13,000 patients disclosed no effects of RAS inhibitors on DR progression in hypertensive patients, but a reduced risk of DR, and increased possibility of DR regression in normotensive patients [186]. In rank order of anti-hypertensive drug classes, the association with risk of DR progression was lowest with ACE inhibitors, followed by Ang II-AT1R blockers, β-blockers, and finally with calcium-channel blockers [186].

While ACE inhibitors show promising results against DR and diabetic macular edema, several safety questions related to increased kinin levels can be raised, such as hypotension, angioedema, and pain associated with inflammation, which are B2R-mediated [192,193,194,195]. Increased kinin levels are also associated with retinal vascular permeability, inflammation, and neovascularization (Figure 2). A decrease in the degradation of endogenous B1R agonist (des-Arg^9^-BK) was also observed in the plasma of patients treated with an ACE inhibitor [196]. In theory, the use of kinin receptor antagonists can overcome the side effects of ACE inhibitors in the retina.

Furthermore, Ang II-AT1R is a potent enhancer of the pro-inflammatory B1R [174,175,176,177] and ACE inhibition ablated B1R expression in diabetic vessels [197], suggesting that B1R acts as an effector of the RAS (Figure 4). Therefore, targeting the RAS (AT1R and ACE) in DR may be a promising approach to prevent the induction and deleterious effects of B1R. Nonetheless, further studies are needed to unveil the exact mechanism(s) and crosstalk with other components of the RAS/KKS (ACE2, AT2R, and MasR) to address the beneficial versus the detrimental effects of the dual pro- and anti-inflammatory role of B2R in retinal disorders. Until these questions are fully answered, targeting B1R in retinal pathologies associated with inflammation and/or vascular alterations remains by far the best asset, with less possible interaction with other axes involved in physiological signaling pathways.

### 7.2. Renin–Angiotensin System in Age-Related Macular Degeneration

The implication of RAS was also reported to contribute to CNV pathogenesis. Indeed, our single-cell RNA seq showed a high expression of ACE in the neovascular AMD arteries and choriocapillaries (Figure 3). Furthermore, prorenin receptor blockade in a murine model of laser-induced CNV exhibited a significant reduction of CNV, macrophage infiltration, and the upregulation of ICAM-1, monocyte chemotactic protein-1, (MCP-1), VEGF, VEGFR1, and VEGFR2 [198]. Moreover, AT1R inhibition pharmacologically or genetically inhibited CNV and macrophage infiltration [198]. VEGF, ICAM-1, and MCP-1 levels, elevated by CNV induction, were significantly suppressed by ACE inhibition, which led to significant suppression of CNV development to the level seen in AT1R-deficient mice [149]. Despite these significant beneficial effects in rodents, antihypertensive drugs (ACE inhibitors and angiotensin receptor blockers) failed to show any positive effects on AMD in humans [199,200,201].

## 8. Conclusions

Inflammatory and neovascular retinal diseases, including DR and AMD, can lead to severe vision loss if left untreated. Current treatments for these pathologies are invasive and can sometimes worsen the pathology. Besides these side effects, many patients do not respond well or become refractory to these treatments, thus there is an urgent need to identify new therapeutic targets and new treatment strategies. Interestingly, the pro-angiogenic, pro-inflammatory, and vasoactive effects of the KKS make it a promising therapeutic target for treating retinal pathologies associated with inflammation and neovascularization. However, KKS targeting needs to be carefully documented before clinical application, as this system is also involved in physiological functions (such as organ blood-flow perfusion and blood coagulation) [4]. To minimize as much as possible the side effects of a complete shutdown of this system that may lead to ischemia and thrombotic events, it is advisable to use a more selective approach by targeting directly kinin receptors in retinal pathologies. Conflicting data are available regarding the implication of B2R in retinal pathologies. This may be related to its important physiological role on the vasculature and the regulation of blood flow. Thus, the inhibition of this receptor may cause unwanted side effects, notably ischemia, and its role in retinal pathology warrants further investigation. In contrast, currently available data strongly support the contribution of B1R in inflammatory and neovascular retinal diseases. Inhibiting the inducible B1R, by topical eye-drop treatment represents a promising noninvasive therapeutic approach in retinal diseases. This is keeping with the finding that B1R acts as an effector of the RAS (Ang II-AT1R) and may subserve its deleterious effects in ocular diseases.
